# Commentary: Neuromuscular and Muscle Metabolic Functions in MELAS Before and After Resistance Training: A Case Study

**DOI:** 10.3389/fphys.2019.01178

**Published:** 2019-09-13

**Authors:** Josef Finsterer

**Affiliations:** Krankenanstalt Rudolfstiftung, Messerli Institute, Vienna, Austria

**Keywords:** MELAS, stroke-like episode, epilepsy, mtDNA, exercise, myopathy, resistance training

## Introduction

Treatment of myopathy in MELAS relies on the application of antioxidants, co-factors, vitamins, the ketogenic diet, and physical exercise. Systematic studies of muscle training in mitochondrial disorders (MIDs) has been only rarely carried out (Porcelli et al., 2016) but a beneficial effect has been reported in most of these studies (Cade et al., 2017). In a recent study article published in Frontiers in Physiology, Venturelli et al. (2019) reported a 21 years old Caucasian male with mitochondrial encephalopathy lactic acidosis and stroke-like episodes (MELAS) syndrome who profited from resistance training at 85% of one repetition maximum by increasing the body mass by 1.4 kg during a training period of 12 weeks. The study raised the following comments and concerns.

## Comments and Concerns

The main shortcoming of the study is that the genetic cause of MELAS was not mentioned in the reported patient. Though the variant m.3243A>G in the tRNA(Lys) gene is the most frequent cause of MELAS, a number of other mtDNA variants may also manifest with the MELAS phenotype (El-Hattab et al., 1993-2019). Since the type of mutation strongly determines the phenotype, it is crucial to confirm the genetic cause of MELAS by appropriate genetic investigations.

A further shortcoming of the study is that heteroplasmy rates of the culprit mtDNA variant were not provided. Knowing heteroplasmy rates from various tissues, such as muscle, lymphocytes, buccal mucosa cells, hair follicles, skin fibroblasts, or urinary epithelial cells is crucial as they may determine the pathogenicity of the variant, the severity of the phenotype, prognosis, and the outcome of the condition.

Misleading is the therapeutic management of systolic dysfunction (Porcelli et al., 2016). Systolic dysfunction and heart failure are not treated by implantation of a pacemaker but by application of angiotensin-converting enzyme inhibitors, beta-blockers, angiotensin-receptor antagonists, diuretics, or levosimendan (Egbuche et al., 2019). We should know if there was systolic dysfunction on echocardiography, if there was leg edema, neck vein distension, or exertional dyspnoea on clinical examination, and if pro brain natriuretic peptide (BNP) values were increased. Knowing cardiac functions is crucial as they determine the load bearing capacity for exercise of an individual and the outcome of the individual.

The beneficial effect of resistance training is dependent on the intellectual abilities and cooperation of the individual patient. Since MELAS frequently goes along with cognitive decline or psychiatric abnormalities (Kraya et al., 2019), it should be mentioned that predominantly patients without cerebral involvement can be selected for the proposed type of physical activity.

A further shortcoming of the study is that no monitoring of oxidative stress parameters, serum lactate levels, or creatine-kinase values was carried out. Since the muscle is frequently affected in MELAS (El-Hattab et al., 1993-2019), excessive exercise may result in further oxidative stress and thus damage of the tissue and consequently in increasing lactate and creatine-kinase values. Changes in muscle morphology and function could be assessed by P-MRS, muscle MR, or muscle biopsy pre-/post exercise.

Missing in this study is the medication the patient was regularly taking. Since many drugs strongly influence muscle performance in MELAS, it is crucial to know which drugs the proband was regularly taking. Particularly from anti-seizure drugs it is well-known that they can be mitochondrion-toxic (Finsterer, 2017). Since patients with MELAS frequently develop epilepsy, we should know if seizures occurred during the period of the intervention.

The patient did not attend 5 of 36 scheduled training sessions. We should know if the reason for skipping the appointments was impaired cognitive abilities, illness, or unwillingness.

Since the patient had dysphagia, it is conceivable that reduced muscle mass was simply due to starving and not attributable to myopathy. We should know if serum protein levels were normal or reduced and if the nutrition profile was normal or not, and if there was renal insufficiency with tubular dysfunction and consecutive proteinurea.

## Discussion

Though the study is interesting and the results promising [increase in body and thigh muscle mass, increased maximal voluntary torque (MVC), improved mVO_2_ kinetics], factors determining the effect of the training should be more strongly considered and discussed. These factors include the type of genetic defect, the heteroplasmy rates, the degree of cardiac involvement, the intellectual capabilities and cooperation of the proband, the drugs the index patient was regularly taking, and if there was malnutrition due to protein loss via the kidneys or the guts. First degree relatives should be investigated and the training effect should be monitored by measuring oxidative stress parameters, ATP production, mitochondrial membrane potential, apoptosis, and the anti-oxidative capacity. It should be also discussed that a disease characterized by fatigue, exercise intolerance, and sleepiness is only accessible to exercise training if the patient is highly motivated and willing to follow the instructions and the overall goal of treating physicians.

## Author Contributions

JF was responsible for design, literature search, discussion, first draft, and critical comments.

### Conflict of Interest Statement

The author declares that the research was conducted in the absence of any commercial or financial relationships that could be construed as a potential conflict of interest.

## References

[B1] Cade W. T. Reeds D. N. Peterson L. R. Bohnert K. L. Tinius R. A. Benni P. B.. (2017). Endurance exercise training in young adults with barth syndrome: a pilot study. JIMD Rep. 32, 15–24. 10.1007/8904_2016_553 27295193 PMC5362555

[B2] Egbuche O. Hanna B. Onuorah I. Uko E. Taha Y. Ghali J. K.. (2019). Contemporary pharmacologic management of heart failure with reduced ejection fraction - a review. Curr. Cardiol. Rev. 10.2174/1573403X15666190709185011. [Epub ahead of print]. PMC7393599 31288726

[B3] El-Hattab A. W. Almannai M. Scaglia F. (1993-2019). MELAS. 2001 Feb 27 [updated 2018 Nov 29], Inin GeneReviews® [Internet: ], eds Adam MP M. P. Ardinger HH H. H. Pagon RA R. A. Wallace SE S. E. Bean LJH L. J. H. Stephens K K. Amemiya A A. editors. GeneReviews® [Internet]. (Seattle, (WA): University of Washington) Seattle; 1993-2019. Available from online at: http://www.ncbi.nlm.nih.gov/books/NBK1233/

[B4] Finsterer J. (2017). Toxicity of antiepileptic drugs to mitochondria. Handb. Exp. Pharmacol. 240: 473–488. 10.1007/164_2016_2 27590225

[B5] Kraya T. Neumann L. Paelecke-Habermann Y. Deschauer M. Stoevesandt D. Zierz S.. (2019). Cognitive impairment, clinical severity and MRI changes in MELAS syndrome. Mitochondrion 44: 53–57. 10.1016/j.mito.2017.12.012 29289801

[B6] Porcelli S. Marzorati M. Morandi L. Grassi B. (2016). Home-based aerobic exercise training improves skeletal muscle oxidative metabolism in patients with metabolic myopathies. J. Appl. Physiol. 121, 699–708. 10.1152/japplphysiol.00885.2015 27445303

[B7] Venturelli M. Villa F. Ruzzante F. Tarperi C. Rudi D. Milanese C.. (2019). Neuromuscular and muscle metabolic functions in MELAS before and after resistance training: a case study. Front. Physiol. 10:503. 10.3389/fphys.2019.00503 31105594 PMC6498991

